# Delay in commencement of palliative care service episodes provided to Indigenous and non-Indigenous patients: cross-sectional analysis of an Australian multi-jurisdictional dataset

**DOI:** 10.1186/s12904-018-0380-7

**Published:** 2018-12-22

**Authors:** John A. Woods, Claire E. Johnson, Hanh T. Ngo, Judith M. Katzenellenbogen, Kevin Murray, Sandra C. Thompson

**Affiliations:** 1 Western Australian Centre for Rural Health, School of Population and Global Health, The University of Western Australia (M706), 35 Stirling Highway, Crawley, WA 6009 Australia; 2 Cancer and Palliative Care Research and Evaluation Unit, Medical School, The University of Western Australia, Murdoch, WA Australia; 3 School of Nursing and Midwifery, Monash University, Clayton, VIC Australia; 4 Eastern Health, Wantirna, VIC Australia; 5 Rural Clinical School, The University of Western Australia, Nedlands, WA Australia; 6 Discipline of Emergency Medicine, Medical School, The University of Western Australia, Nedlands, WA Australia; 7 School of Population and Global Health, The University of Western Australia, Nedlands, WA Australia; 8 Telethon Kids Institute, The University of Western Australia, Nedlands, WA Australia

**Keywords:** Oceanic ancestry group, Aboriginal, Palliative care, End-of-life care, Episode of care, Time-to-treatment, Healthcare disparities, Health services research, Cross-sectional studies

## Abstract

**Background:**

Rapid effective responsiveness to patient needs is pivotal to high quality palliative care. Aboriginal and Torres Strait Islander (Indigenous) people are susceptible to life-limiting illnesses at younger ages than other Australians and experience inequity of health service provision. The Palliative Care Outcomes Collaboration collects Australia-wide health service data on patient care, and has established performance benchmarks for specialist palliative care services. We investigated whether the benchmark for timely commencement of palliative care episodes (occurrence of delay >1 day after being designated ‘ready for care’ in <10% instances) is being met for Indigenous Australians in participating services. Additionally, we investigated the association between identification as Indigenous and delay.

**Methods:**

Using multi-jurisdictional Palliative Care Outcomes Collaboration data, this cross-sectional analytical study investigated all episodes of care (*n* = 84,238) provided to patients ≥18 years (*n* = 61,073: Indigenous *n* = 645) in hospital and community settings commenced and completed during the period 01/07/2013–30/06/2015. Proportions of episodes resulting in delay were determined. Crude and adjusted odds of delay among Indigenous compared with non-Indigenous patients were investigated using multiple logistic regression, with missing data handled by multiple imputation.

**Results:**

The benchmark was met for both Indigenous and non-Indigenous patients (delay in 8.3 and 8.4% episodes respectively). However, the likelihood of delay was modestly higher in episodes provided to Indigenous than non-Indigenous patients (adjusted odds ratio [aOR], 1.41; 95% confidence interval [CI] 1.07–1.86). Excess delay among Indigenous patients was accentuated in first episodes (aOR, 1.53; 95% CI 1.14–2.06), in patients aged < 65 years (aOR, 1.66; 95% CI 1.14–2.41), and among those residing in Inner Regional areas (aOR, 1.97; 95% CI 1.19–3.28), and also approached significance among those in outer regional, remote and very remote areas collectively (aOR, 1.72; CI 0.97–3.05).

**Conclusions:**

Although the timeliness benchmark is being met for Indigenous Australians in palliative care, they may experience delayed initiation of care episodes, particularly if younger, and especially at first encounter with a service. Qualitative research is required to explore determinants of delay in initiating palliative care episodes. The timeliness of initial referral for specialist palliative care in this population remains to be determined.

**Electronic supplementary material:**

The online version of this article (10.1186/s12904-018-0380-7) contains supplementary material, which is available to authorized users.

## Background

Aboriginal and Torres Strait Islander (hereafter respectfully referred to as Indigenous) people experience shorter life expectancies than other Australians, with higher age-standardised mortality rates from neoplasms and other life-limiting conditions [1]. Furthermore, Indigenous Australians experience disparities in health service provision across a range of settings due to cross-cultural misunderstandings and other institutional barriers to culturally safe care [2]. For many, geographical remoteness and logistical problems impede health service access, accentuating poorer outcomes [3, 4]. Substantial qualitative research addresses the needs, preferences and experiences of Indigenous Australians in end-of-life care settings [5, 6]. However, equity of care in this context has not been investigated quantitatively or across jurisdictions.

The Palliative Care Outcomes Collaboration (PCOC) is an Australian Commonwealth Government-funded program established in 2005 to improve service quality by (i) embedding standardised clinical assessment tools into routine clinical practice and (ii) systematic point-of-care data collection for reporting, benchmarking and feedback to service providers [7] ‘to support care planning and drive improvements in palliative care’ [8]. By 2010, organisations accounting for more than 80% of specialist palliative care service provision nationwide were submitting data to PCOC on inpatient, outpatient and community care [7].

A capacity to respond rapidly and effectively to the changing needs of patients (and their carers) is fundamental to the quality of palliative care [9]. Following extensive consultation with service providers, PCOC developed national quality-of-care benchmarks [7, 8]. Among these, the timeliness of care benchmark stipulates that ‘90% of patients must have their episode commence on the day of or the day after [being deemed] ready for care’ [8]. It must be emphasised that the benchmark measure investigated in this study is a ‘downstream’ measure of service quality, pertaining to patients already accepted for palliative care by a specialist service. The benchmark is predicated on the understanding that a patient’s need for specialist palliative interventions may be intermittent and recurrent. Accordingly, the benchmark reflects timeliness of initiation of each care episode provided to a patient by a service, rather than only at the patient’s initial acceptance for care by the service. It does not address the time interval between when a patient first becomes ready to benefit from palliative care, is recognised as such by treating clinicians, and is referred appropriately to and accepted by a specialist palliative care service.

Using PCOC data, we examined timeliness of commencing an episode of care against the benchmark, as part of a research project investigating palliative care quality provided to Indigenous compared with non-Indigenous patients in participating services.

## Methods

### Study design, data source and sample

**Fig. 1:**
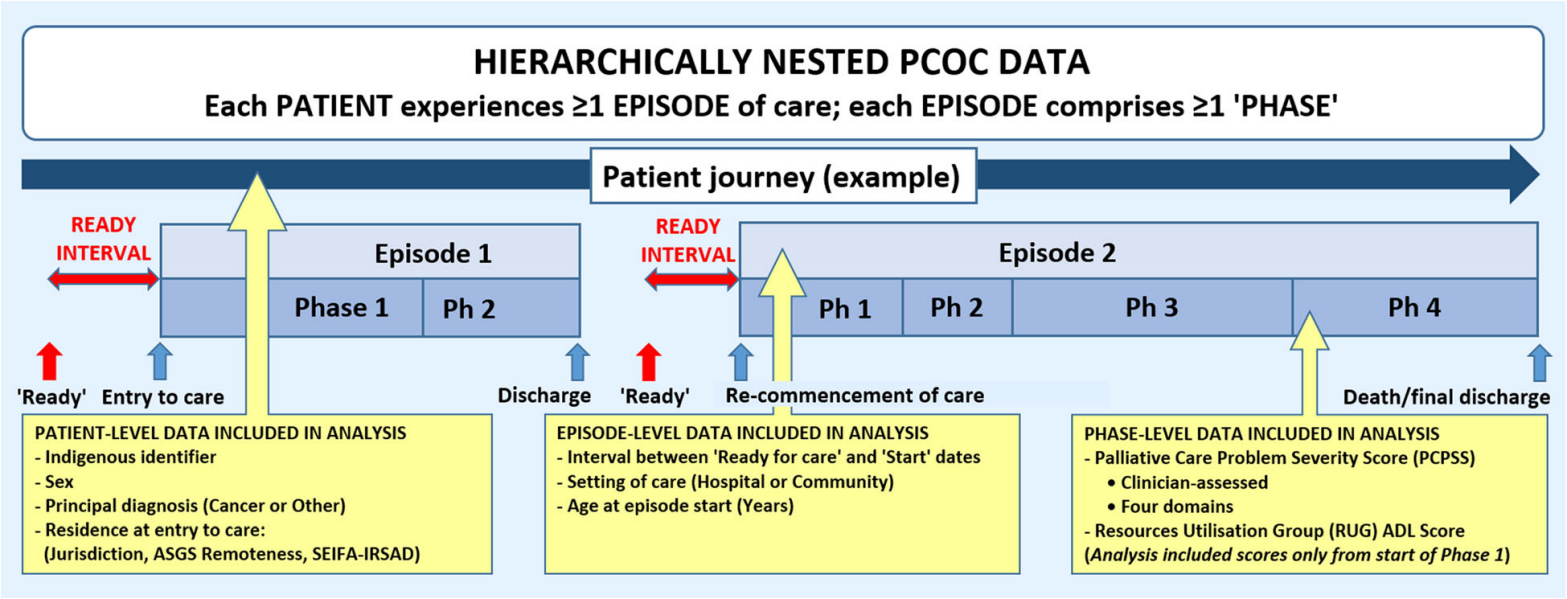
Structure of PCOC dataset, highlighting the ‘ready interval’. Legend: ASGS: Australian Statistical Geography Standard; Phase/Ph: Clinical phase of care; SEIFA-IRSAD: Socio-Economic Index for Areas–Index of Relative Socio-Economic Advantage and Disadvantage. Source: [33]

This cross-sectional analytical study used de-identified nationwide PCOC data, with ‘episode-of-care’ as the unit of analysis. The PCOC dataset is nested hierarchically, comprising (i) personal details captured once at service entry to care, (ii) data pertaining to each episode-of-care the patient receives, and (iii) data recorded at the beginning and end of one or more clinical ‘phases’ within each episode (Fig. 1) [8, 10]. Patients were distinguished by a numeric code *within* a service but their care could not be tracked between services. ‘Ready for care’ data were not recorded consistently across participating services until mid-2013. This study comprised all hospital-based (overnight admissions, day admissions and ambulatory care) and community-based episodes of care provided to adult patients (≥18 years at episode start) in participating services throughout Australia, commenced and completed between 1-July-2013 and 30-June-2015.

### Variables

The benchmark was operationalised by collapsing the calendar interval between a patient’s ‘ready for care’ date and the episode start date (Fig. 1) into a binary outcome variable (> 1 day versus 0–1 day), with a ‘delay’ in initiating care defined as an interval of > 1 day. Intervals considered implausible (> 30 days [*n* = 259]; reversed [i.e., episode commencement before ‘ready for care’ date: *n* = 45]; total: 304 = 0.36%) were recoded as missing.

The principal explanatory variable was the Indigenous identifier, based on data recorded once for each patient at entry to a service. For this study, patients were categorised as Indigenous, (if originally coded as Aboriginal and/or Torres Strait Islander), non-Indigenous or Identifier Missing.

Other person-level variables were sex, principal diagnosis, and those based on residence at entry to care. Principal diagnosis was collapsed into a binary variable (cancer/other) from more detailed organ/system-specific diagnostic code [11]. Values based on implausible combinations of these variables (e.g., female with prostate cancer, male with gynaecological cancer) (*n* = 97 episodes [0.12%]) were recoded as missing. National population quintiles of the Socio-Economic Index for Areas Index of Relative Socioeconomic Advantage and Disadvantage (SEIFA-IRSAD) [12] provided a census-based area-level measure of social disadvantage based on patients’ residence. Remoteness of residence according to the Australian Statistical Geography Standard (2011) [13] was collapsed from five categories into four: Major Cities, Inner Regional, Outer Regional and Remote/Very Remote. Jurisdiction of residence categories comprised the six Australian states, with no data provided by services from the Northern Territory (population ~ 230,000; of whom approximately 69,000 [30%] identify as Indigenous) [14]. Data from the single Australian Capital Territory service were combined with New South Wales data to preserve service confidentiality.

Data ascertained at the beginning of each episode were start date (collapsed into two one-year calendar periods, to capture evolution in the quality of a newly instituted measure of care), episode sequence (first/subsequent), setting, age (years, modelled in both continuous and binary form [< 65 versus ≥65 years]), and phase-based measures of patients’ clinical and functional status. In order to sequence episodes of care as accurately as possible, sequencing was optimised by lookback either to PCOC inception in 2005 or as far as possible from the data of each participating service. Setting of care was categorised as admitted overnight, hospital ambulatory/day-case admission or outpatient visit, and community-based. Setting was collapsed as a binary variable (hospital/community): hospital ‘admitted overnight’ episodes were merged with a small number (*n* = 907 [1.1%]) of day-admission/outpatient cases.

Data on end-of-life-related problems and functional status are recorded at various time-points during an episode. The current analysis included only start-of-episode scores. Problem status was determined from the Palliative Care Problem Severity Score (PCPSS), a validated clinician-rated score comprising four domains (pain, other symptoms, psychological/spiritual and family/carer problems) [15]. Scores from each domain were collapsed into binary form (absent/mild versus moderate/severe) and analyzed as separate explanatory variables. Similarly, data on patient’s functional status from the Resources Utilisation Group–Activities of Daily Living (RUG-ADL) total score [16] were collapsed into approximate sample-based tertiles (1 = most independent, 2 = intermediate, 3 = most dependent).

All variables incorporated into the analysis had missing values, with the exception of episode start date, episode sequence number, and episode setting. The multiple imputation model (see below) also incorporated ‘auxiliary’ variables from the PCOC dataset, i.e., variables that were themselves not included as covariates in analysis but were shown to be associated with the values and ‘missingness’ patterns of covariates with missing values (and could thereby be used to optimise imputation of these missing data) [17]. Examples from our analysis are the nominal variables ‘accommodation at episode start’ and ‘referral source’.

### Statistical analysis

Baseline (person-level) and episode-level characteristics of Indigenous and non-Indigenous patients were compared, with significance determined by χ^2^ and t-tests. Determinants of delay were investigated using univariate and multiple logistic regression models accounting for unbalanced clustering of episode-based measures in individual subjects. Multicollinearity of covariates was examined by investigation of variance inflation, and the numeric covariate ‘age’ was log-transformed to account for non-linearity. First-order interactions between covariates were included on criteria of combined a priori plausibility and statistical significance (*p* < 0.01). No significant interaction between the Indigenous identifier and any other variable was detected.

Missing values across variables incorporated in the analyses were quantified. Analyses were performed on both (i) the complete case dataset comprising records for which no missing values were recorded for any variable incorporated in the model, and (ii) a dataset with missing values handled by multiple imputation (MI).

MI involves generating a set of plausible values for missing data across one or more variables in a dataset, based on relationships between distributions of different variables in observed data, in a manner that accounts appropriately for uncertainty. By this means, multiple datasets are created from the original dataset, each with a complete set of values for variables modelled in the imputation process. In each of these created datasets, each originally missing value has been replaced with a value drawn randomly from the set of estimates applicable to each variable. Analyses are then performed identically across each dataset. The individual parameter estimates obtained from each dataset are then combined to produce overall statistics [17].

Substantive Model Compatible Fully Conditional Specification was adopted as the basis for imputation, given the need to incorporate transformed variables and interaction terms compatible across the imputation and the analytic models [18]. Algorithm convergence across *n* = 10 iterations was checked by inspection of pseudo-time-series plots of imputed variables [19], m = 15 datasets were imputed, and the Monte Carlo errors of generated estimates were investigated to determine their reproducibility [17].

The odds ratio (OR) of delay associated with the Indigenous identifier was estimated without and with adjustment for potential confounding patient-level and episode-level variables, including palliative care problems and functional status at episode start. The association of the Indigenous identifier with delay was investigated for the full sample and across various subgroups. The subgroups for analysis were chosen on the basis of a priori plausibility and/or demonstration that the covariate for subgrouping (e.g., age group, setting of care) was independently associated with delay.

As sensitivity analyses, odds ratio estimates calculated from the multiple imputation data were compared with those calculated from the ‘complete case’ data.

Additionally, the calendar pattern in proportion of episodes resulting in delay was investigated by estimating proportions of delay by month based on the multiple imputation data, in hospital and community settings separately, for episodes of all patients and also by Indigenous identifier.

All analyses were conducted using Stata 13 (StataCorp, College Station, Texas, USA).

### Ethics

Ethics approval for the study was provided by the Western Australian Aboriginal Health Ethics Committee (reference, 616) and the University of Western Australia Human Research Ethics Committee (reference, RA/4/1/7441).

## Results

### Characteristics of patients and episodes of care

During the study period, 84,238 episodes of care were experienced by 61,073 patients (mean 1.38; range 1–19) (Table 1). Data on one or more variables were missing from 10,581 records (12.6%). Indigenous patients were younger on average than their non-Indigenous counterparts, and more likely to be female and/or live in an area of socioeconomic disadvantage. Those with Indigenous identifier missing were demographically more similar to non-Indigenous than to Indigenous patients. Cancer rather than a non-neoplastic illness was the principal diagnosis in a majority of both groups (Table 1).

**Table 1:** Characteristics of patients and their palliative care episodes by Indigenous Identifier, with indication of missing data

	Non-Indigenous		Indigenous		Identifier missing		Total	
	N	(%)	N	(%)	N	(%)	N	(%)
Patient-based characteristics								
Total Patients	59,226	(97.0)	645	(1.1)	1202	(2.0)	61,073	(100.0)
Sex								
Female	27,740	(46.8)	337	(52.3)	551	(45.8)	28,628	(46.9)
Male	31,482	(53.2)	308	(47.8)	650	(54.1)	32,440	(53.1)
Missing	4	(0.0)	0	(0.0)	1	(0.1)	5	(0.0)
Principal Diagnosis								
Cancer	45,704	(77.2)	481	(74.6)	987	(82.1)	47,172	(77.2)
Non-cancer	13,212	(22.3)	162	(25.1)	203	(16.9)	13,577	(22.2)
Missing	310	(0.5)	2	(0.3)	12	(1.0)	324	(0.5)
Remoteness^a^								
Major Cities	45,351	(76.6)	336	(52.1)	871	(72.5)	46,558	(76.2)
Inner Regional	9985	(16.9)	161	(25.0)	285	(23.7)	10,431	(17.1)
Outer Regional	3177	(5.4)	108	(16.7)	37	(3.1)	3322	(5.4)
Remote/Very Remote	293	(0.5)	39	(6.1)	4	(0.3)	336	(0.6)
Missing	420	(0.7)	1	(0.2)	5	(0.4)	426	(0.7)
SEIFA^b^ (quintile)								
1 (most disadvantaged)	8849	(14.9)	200	(31.0)	182	(15.1)	9231	(15.1)
2	8369	(14.1)	125	(19.4)	150	(12.5)	8644	(14.2)
3	11,661	(19.7)	148	(23.0)	287	(23.9)	12,096	(19.8)
4	12,727	(21.5)	104	(16.1)	230	(19.1)	13,061	(21.4)
5 (least disadvantaged)	17,161	(29.0)	67	(10.4)	346	(28.8)	17,574	(28.8)
Missing	459	(0.8)	1	(0.2)	7	(0.6)	467	(0.8)
Jurisdiction of Residence								
NSW	12,774	(21.6)	158	(24.5)	165	(13.7)	13,097	(21.4)
QLD	13,119	(22.2)	171	(26.5)	226	(18.8)	13,516	(22.1)
SA	4375	(7.4)	43	(6.7)	215	(17.9)	4633	(7.6)
TAS	2645	(4.5)	48	(7.4)	28	(2.3)	2721	(4.5)
VIC	16,740	(28.3)	67	(10.4)	176	(14.6)	16,983	(27.8)
WA	9207	(15.6)	156	(24.2)	388	(32.3)	9751	(16.0)
Missing	366	(0.6)	2	(0.3)	4	(0.3)	372	(0.6)
Episode-based characteristics								
Total episodes	81,571	(96.8)	983	(1.2)	1684	(2.0)	84,238	(100.0)
Age group^c^								
< 65 years	21,722	(26.6)	535	(54.4)	506	(30.1)	22,763	(27.0)
≥ 65 years	59,842	(73.4)	448	(45.6)	1178	(70)	61,468	(73.0)
Missing	7	(0.0)	0	(0.0)	0	(0.0)	7	(0.0)
Setting								
Hospital	46,405	(56.9)	620	(63.1)	778	(46.2)	47,803	(56.8)
Community	35,166	(43.1)	363	(36.9)	906	(53.8)	36,435	(43.3)
Missing	0	(0.0)	0	(0.0)	0	(0.0)	0	(0.0)
Palliative care problems (at episode start)^d^								
Pain								
Absent-Mild	59,463	(72.9)	653	(66.4)	1187	(70.5)	61,303	(72.8)
Mod-Severe	20,508	(25.1)	306	(31.1)	420	(24.9)	21,234	(25.2)
Missing	1600	(2.0)	24	(2.4)	77	(4.6)	1701	(2.0)
Other symptoms								
Absent-Mild	46,436	(56.9)	542	(55.1)	902	(53.6)	47,880	(56.8)
Mod-Severe	32,587	(40.0)	408	(41.5)	684	(40.6)	33,679	(40)
Missing	2548	(3.1)	33	(3.4)	98	(5.8)	2679	(3.2)
Psychological/spiritual								
Absent-Mild	60,050	(73.6)	687	(69.9)	1154	(68.5)	61,891	(73.5)
Mod-Severe	20,329	(24.9)	275	(28.0)	497	(29.5)	21,101	(25.1)
Missing	1192	(1.5)	21	(2.1)	33	(2.0)	1246	(1.5)
Family/carer								
Absent-Mild	51,955	(63.7)	604	(61.4)	1029	(61.1)	53,588	(63.6)
Mod-Severe	26,657	(32.7)	285	(29.0)	580	(34.4)	27,522	(32.7)
Missing	2959	(3.6)	94	(9.6)	75	(4.5)	3128	(3.7)
Functional Status – episode start (approx. tertiles)^e^								
1 (most independent)	28,359	(34.8)	348	(35.4)	682	(40.5)	29,389	(34.9)
2	25,922	(31.8)	333	(33.9)	540	(32.1)	26,795	(31.8)
3 (least independent)	26,203	(32.1)	291	(29.6)	434	(25.8)	26,928	(32.0)
Missing	1087	(1.3)	11	(1.1)	28	(1.7)	1126	(1.3)
Episode sequence^f^								
First	25,977	(31.9)	378	(38.5)	558	(33.1)	26,913	(32.0)
Subsequent	55,594	(68.2)	605	(61.6)	1126	(66.9)	57,325	(68.1)
Missing	0	(0.0)	0	(0.0)	0	(0.0)	0	(0.0)
Calendar period								
Jul 2013–Jun 2014	41,054	(50.4	507	(51.6)	855	(50.7)	42,416	(50.4)
Jul 2014–Jun 2015	40,517	(49.7	476	(48.4)	829	(49.2)	41,822	(49.7)
Missing	0	(0.0)	0	(0.0)	0	(0.0)	0	(0.0)

^a^Australian Statistical Geography Standard (ASGS) [13] recorded at entry to first episode of care

^b^Socio-Economic Index for Areas (SEIFA) Index of Relative Socio-Economic Advantage and Disadvantage (IRSAD) [12] recorded at entry to first episode of care

^c^Recorded at entry to first episode of care

^d^Pain, Other symptoms, Psychological/Spiritual and Family/Carer are the four domains of the clinician-rated Palliative Care Problem Severity Score (PCPSS) [15]

^e^Resource Utilisation Groups–Activities of Daily Living (RUG-ADL) total score [16]

^f^Sequence based on lookback to inception service’s participation in PCOC collection

The majority of episodes occurred in a hospital rather than a community setting, with Indigenous patients more likely to receive hospital-based care. Indigenous patients were more likely than non-Indigenous patients to be clinician-assessed as having moderate-severe (rather than absent-mild) levels of pain and psychological/spiritual distress at episode start. However, the two groups exhibited similar proportions of moderate-severe level ‘other’ physical symptoms and family/carer problems. Functional status at episode start did not differ significantly between the groups (Table 1).

### Delay in commencing care

Overall, the benchmark was met for both the Indigenous and non-Indigenous patient groups, with a near-identical raw likelihood of delay (Additional file 1: Table S1). The mean interval between being identified as ready and episode start was 0.54 days overall (episodes among Indigenous patients 0.57 days versus non-Indigenous 0.54 days; *p* = 0.67).

**Fig. 2:**
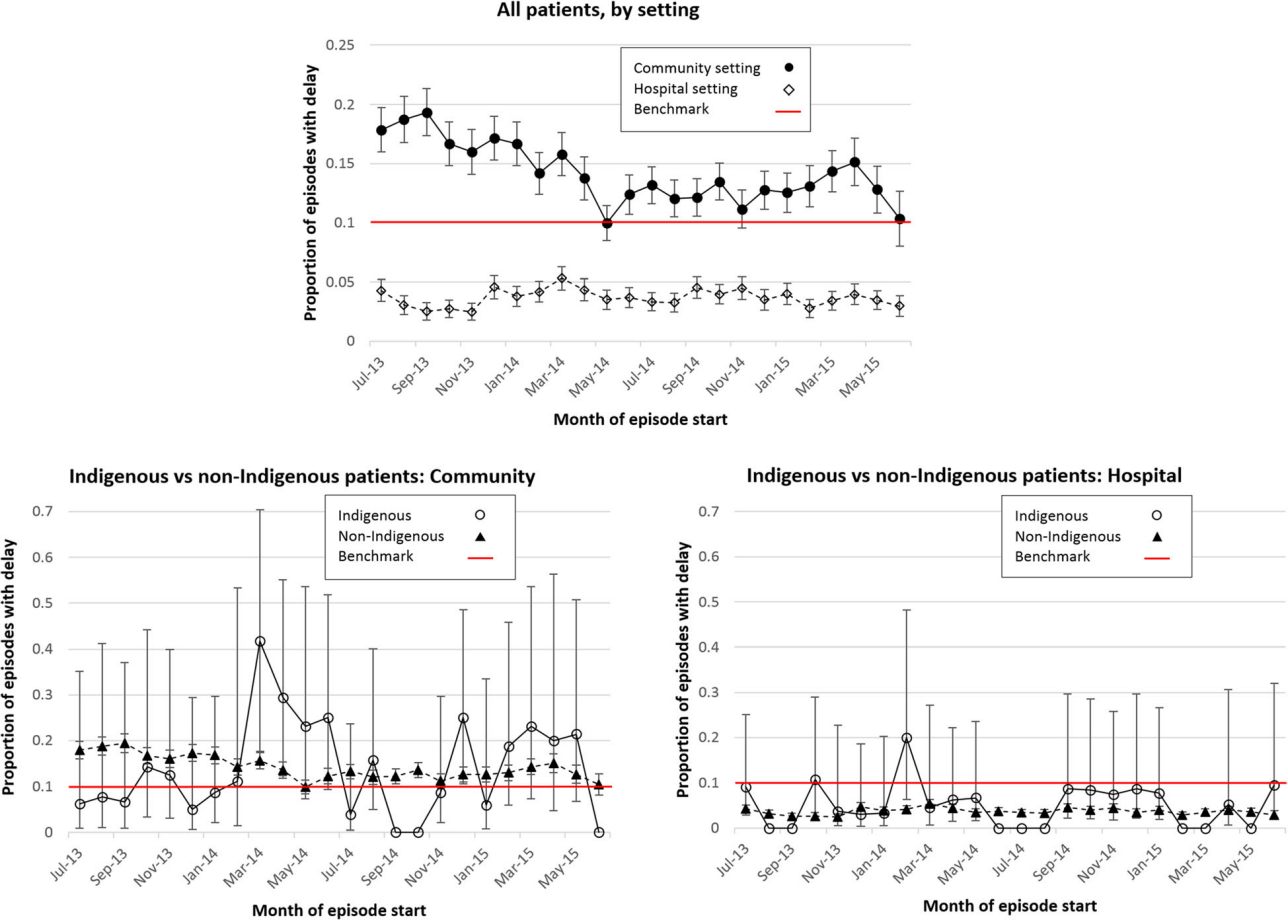
Calendar trends: proportion of episodes with delay. Multiple imputation data estimates with 95% confidence bands. Confidence bands are not displayed for months in which the proportion equals zero

Based on estimates from the multiple imputation data for all patients combined, the proportion of hospital-based episodes with delay was well within the benchmark during all of the twenty-four months of the study, and this proportion did not shift appreciably over the calendar period. In community settings, the proportion of episodes associated with delay for all patients combined diminished progressively during the first year of the study period, with no clear trend in the second year (Fig. 2a). Monthly estimates of proportions of episodes among Indigenous patients were too unstable to discern calendar trends (Figs. 2b and c).

After multiple imputation modelling and adjustment for potential confounders, episodes among Indigenous patients were associated with significantly higher odds of delay (adjusted odds ratio [aOR] from multiple imputation model: 1.41 [CI 1.07–1.86]) (Table 2). Delay was not associated with the patients’ age, sex or principal diagnosis category (Additional file 1: Table S1). Patients resident in Inner Regional and Outer Regional areas had lower odds of delay than their counterparts residing in Major Cities, whereas the odds were higher among those from Remote or Very Remote areas. There was no clear pattern of variation in delay across quintiles of social disadvantage. The setting of care was strongly associated with delay, which occurred in community settings more frequently than in hospitals. Further, delay was more likely in subsequent compared with first episodes, with this difference accounted for entirely by episodes in the community setting, where the likelihood of delay increased from 11.6 to 19.3% between first and subsequent episodes. Moderate/severe (in comparison with absent/mild) levels of clinician-assessed pain and ‘other’ physical symptoms were associated with lower odds of delay, but no differences were evident for family or psychological/spiritual distress. The RUG-ADL score was also associated with delay, which was most frequent among patients in the most-independent tertile, and least frequent among those in the least-independent. The overall odds of delay diminished progressively over the two-year calendar period.

**Table 2:** Association between being identified as Indigenous and delay in commencement of palliative care episodes – sensitivity analysis comparing estimates based on complete case and multiple imputation data

Model	Indicative N^a^	Complete case analyses^b^			Multiple imputation analyses^b^		
		OR^c^	95% CI^d^	*P* value	OR^c^	95% CI^d^	*P* value
(a) Crude (univariate) estimates							
Total sample	73,657/84,238	0.99	0.72–1.36	0.96	1.07	0.80–1.42	0.66
(b) Adjusted estimates, main effects^e^ plus interaction terms^f^							
Total sample	73,657/84,238	1.32	0.98–1.77	0.07	1.41	1.07–1.86	0.02
Restriction by setting (adjusted model)^g^							
Hospital	41,283/47,803	1.35	0.80–2.25	0.26	1.49	0.96–2.32	0.08
Community	32,374/36,435	1.32	0.91–1.91	0.15	1.29	0.91–1.85	0.16
Restriction by episode sequence (adjusted model)^g^							
First episode of care in PCOC records	50,229/57,325	1.42	1.02–1.96	0.04	1.53	1.14–2.06	0.005
Second or subsequent episode of care in PCOC records	23,428/26,913	1.03	0.55–1.94	0.93	1.08	0.61–1.90	0.79
Restriction by age group (adjusted model)^g^							
< 65 years at episode start	19,737/~ 22,767	1.52	0.98–2.36	0.06	1.66	1.14–2.41	0.009
≥ 65 years at episode start	53,920/~ 61,471	1.14	0.75–1.74	0.54	1.09	0.71–1.67	0.69
Restriction by remoteness of residence (adjusted model)^g^							
Major city	55,592/~ 63,850	1.09	0.72–1.65	0.70	1.16	0.77–1.73	0.48
Inner Regional	13,514/~ 15,069	2.15	1.24–3.73	0.005	1.97	1.19–3.28	0.009
Outer Regional/Remote/Very Remote	4551/~ 5319	1.18	0.57–2.44	0.66	1.72	0.97–3.05	0.06
Restriction by calendar period (adjusted model)^g^							
Jul 2013 – Jun 2014	36,850/42,416	1.28	0.84–1.95	0.24	1.38	0.94–2.02	0.10
Jul 2014 – Jun 2015	36,807/41,822	1.34	0.89–2.01	0.16	1.40	0.95–2.06	0.09
Restriction by principal diagnosis category (adjusted model)^g^							
Cancer	59,455/~ 67,680	1.34	0.97–1.85	0.08	1.41	1.04–1.91	0.03
Other	14,202/~ 16,558	1.23	0.60–2.50	0.58	1.42	0.78–2.59	0.25

^a^Numbers shown are those from Complete Case analyses/Multiple imputation analyses. For the multiple imputation analyses, the number displayed for total sample is the actual number of episodes in the analyses; for stratified subgroups of variables with missing values, the distribution of numbers of episodes across subgroups varies slightly between imputed datasets because of the imputation process. In such cases (marked ~), indicative numbers are shown from dataset #1 of m = 15 imputed datasets. (There were zero missing values for setting, episode sequence and calendar period [Table 1])

^b^All estimates based on logistic regression models accounting for unbalanced clustering of episodes among individual patients

^c^OR: Odds ratio for episodes in Indigenous versus non-Indigenous patients

^d^CI: Confidence interval

^e^Covariates in multiple logistic regression: age in years (continuous), sex, principal diagnosis category (cancer/other), episode setting (community/ hospital [overnight & same-day admissions plus outpatients]), remoteness of residence at baseline, social disadvantage of area of residence at baseline, episode of care sequence (based on lookback to inception of service’s participation in PCOC collection), jurisdiction, calendar month, clinical status at episode start (four domains of PCPSS modelled separately), functional status at episode start (RUG-ADL total score modelled by tertile). Data breakdown by individual jurisdiction not presented

^f^Interactions included in analyses of the total sample were: settingXsequence, calendar-periodXsetting, jurisdictionXsequence, jurisdictionXsetting, jurisdictionXseifa, sequenceXremoteness, seifaXremoteness. An applicable subset of these interactions was selected for each restricted analysis

^g^Subgroup estimates are adjusted for all potential confounding factors (listed in [e,f] above) other than the covariate (main effect and interaction terms if applicable) by which the analysis is restricted. In the analyses restricted by age group, age in years as a continuous variable remained in the model

In subgroup analyses (Table 2), a higher odds of delay commencing care in episodes experienced by Indigenous compared with non-Indigenous patients was evident in first but not subsequent episodes of care, in the under-65 years age-group but not the group aged ≥65 years, and among patients resident in Inner Regional areas but not those from Major Cities. The disparity between episodes among Indigenous and non-Indigenous patients was similar whether the principal diagnosis was cancer or ‘other’ diseases, although the difference reached statistical significance only in the cancer group as a consequence of larger sample size.

## Discussion

Overall, the benchmark for timeliness in initiation of an episode of palliative care appears to have been met for both Indigenous and non-Indigenous Australian patients who were provided care by specialist services contributing data to PCOC. However, our findings suggest that, after adjustment for potential confounding factors, Indigenous Australians are modestly more likely than other patients to experience delay between being designated ready for care and commencing the episode of care. Disproportionate delay was evident among Indigenous patients who were younger (< 65 years), resident outside major cities, and/or experiencing a first (as opposed to subsequent) episode of care within a service.

There is a burgeoning literature on equity in timeliness of access to palliative care in general, including published data on ethnic/racial disparities [20, 21]. Furthermore, although published data are scanty, differences in service provision between first and subsequent episodes of home/community health care have been highlighted previously [22]. It is increasingly recommended that expert palliative care should be initiated as early as possible following the diagnosis of a life-limiting illness (i.e., such that concurrent disease-modifying and palliative management is appropriate in many circumstances) [23, 24]. Internationally, it is recognised that persons from disadvantaged and minority ethnic populations often fail to receive timely initial referral [20]. Unfortunately, timeliness of initial acceptance for care by a specialist palliative care service could not be directly ascertained from these within-service data. Findings from the current study on disparities affecting Indigenous patients are not readily applicable to the context of initial referral, as the spectrum of barriers responsible may differ. For example, shortcomings of institutions in providing culturally safe care, ineffective communication between healthcare providers and Indigenous patients, other psychological barriers (taboos on broaching end-of-life issues) and logistical problems (accommodation, transport) may not be equivalent among patients eligible for and those already receiving palliative care.

PCOC has compiled a large, detailed, high-quality multi-jurisdictional dataset, facilitating richly contextualised palliative care research. However, data shortcomings may have compromised the validity of the findings. We made every effort to circumvent spurious precision of estimates resulting from failure to account for repeated measures in the same individual, and used multiple imputation to reduce potential bias and diminution of power arising from missing data [25] including Indigenous identifiers. Missing or incorrect ascertainment of a patient’s identification as Indigenous is known to hinder health research on Indigenous populations in developed countries [26] Misclassification of reported values across other variables may also have resulted in residual confounding and biased estimates. The inability to track patients cared for across more than one service is likely to have resulted in some double/multiple counting. Some indices measured once at entry to care by a service may be misleading, for example, reported residence may not reflect accommodation at episode start, and SEIFA scores are an ecological rather than an individual/household-level measure of socio-economic disadvantage.

Our aim has been to determine overall national patterns in equity of care in relation to the benchmark. Clearly, single, ‘whole-of-nation’ odds ratio estimates obscure heterogeneity between individual service organisations and between state jurisdictions. Individual services were not distinguishable from the dataset, and the authors have not been permitted to publish service quality data broken down by state. In any case, the small total number of care episodes among Indigenous patients constrains the interpretability of state-level estimates.

Although the dataset is multi-jurisdictional and encompasses the large majority of specialist palliative care services nationwide, it does not capture an unbiased representation of the total. Firstly, there is an under-representation of community-based services, particularly from NSW, the most populous state. Notably, the overall proportion and geographical distribution of persons identifying as Indigenous who have accessed specialist palliative care in this study are discordant with national Census data [27]. Given that > 2.5% of the Australian population identified as Aboriginal and/or Torres Strait Islander in the 2011 and 2016 Census surveys [14, 27], the 1.1% identified in this analysis suggests that disproportionately few Indigenous patients with life-limiting illnesses were cared for by participating services, over and above incorrect or missing Indigenous identification within the dataset. Further, the residential distribution of Indigenous patients in the dataset reflects under-representation of Indigenous patients from non-metropolitan areas, which is particularly marked among those from Remote and Very Remote areas [14]. A small proportion of this discrepancy is plausibly attributable to short-term shift in residence for care of a life-limiting illness prior to entry to the dataset. Furthermore, the Northern Territory, which among Australian jurisdictions has the highest proportion (although not the highest absolute number) of Indigenous residents, contributed no service data during the period of analysis. Accordingly, generalisability of the findings is constrained by under-representation of persons from remote Indigenous Australian communities, who tend to have the highest incidence of [28] and mortality [29] from cancers and other life-limiting disorders [30], and who also experience the poorest access to health services, particularly specialist care.

The nature of the detected disparities could be further clarified through qualitative investigation of the lived experience of Indigenous Australians with life-limiting illness and their carers, particularly to determine the extent to which existing recommendations for service delivery are being met. The Living Model of service delivery to Indigenous Australians in this context [31] incorporates effective communication, attention to psychological issues and practical barriers to care, education, involvement and support of family members, and an emphasis on cultural safety, all of which potentially facilitate timely commencement of care.

Further analyses of PCOC health outcome benchmarks (such as those related to symptom control) [8] in this population is being undertaken as part of the current project. Future research on the timeliness, other measures of quality, and coverage of palliative service care provision to Indigenous Australians and similar populations could be greatly facilitated and enriched by data linkage between palliative services (as has since been implemented by PCOC) and further with other health data sources (hospitals, primary care providers, and disease registries).

## Conclusions

This is the first quantitative multi-jurisdictional investigation of health service quality for Indigenous Australians in palliative care. The dual vulnerability experienced by socially disadvantaged persons with a life-limiting illness [32] underscores the importance of investigating equity of palliative care service provision. Our findings provide reassurance that timeliness of episode-by-episode care provision to Indigenous Australians within the majority of specialist palliative care services nationwide that provide data to PCOC has met the established benchmark overall, while suggesting modest disparities affecting this population. However, these findings do not elucidate between-service heterogeneity in performance and under-represent Indigenous Australians, especially those who reside in the most remote communities nationwide and are thereby likely to be both particularly vulnerable to inequity of health service provision and among the most likely to require end-of-life care.

## Additional file

Additional file 1: **Table S1.** Factors associated with delay (> 1 day) in commencing an episode of care. Percentage of episodes with delay (≥1 day), by category of each determinant, based on raw data. Crude and adjusted odds ratios for delay (≥1 day): sensitivity analyses of complete case compared with multiple imputation data. (XLSX 18 kb)

## Abbreviations

aOR: adjusted odds ratio
ASGS: Australian Statistical Geography Standard
CI: Confidence interval
MI: multiple imputation
NSW: New South Wales
OR: Odds ratio
PCOC: Palliative Care Outcomes Collaboration
PCPSS: Palliative Care Problem Severity Score
QLD: Queensland
RUG-ADL: Resources Utilisation Group–Activities of Daily Living
SA: South Australia
SEIFA-IRSAD: Socio-economic Index for Areas–Index of Relative Social Advantage and Disadvantage
TAS: Tasmania
VIC: Victoria
